# Effect of aging on dimensional accuracy and color stability of CAD-CAM milled and 3D-printed denture base resins: a comparative in-vitro study

**DOI:** 10.1186/s12903-024-04848-9

**Published:** 2024-09-26

**Authors:** Marwa A. Gad, Ahmed M. Abdelhamid, Mahmoud ElSamahy, Salma Abolgheit, Kenda I. Hanno

**Affiliations:** 1 Department of Prosthodontics, Faculty of Dentistry, Alexandria University, Alexandria, Egypt; 2 Department of Dental Biomaterials, Faculty of Dentistry, Alexandria University, Alexandria, Egypt

**Keywords:** CAD-CAM, 3D-printed, Dimensional accuracy, Color stability, Thermocycling, Cleanser

## Abstract

**Background:**

There is a lack of studies comparing the dimensional accuracy and color stability of denture base resins made using computer-aided design and computer-aided manufacturing (CAD-CAM) milling, 3-dimensional (3D) printing, and conventional denture processing techniques. This makes it challenging to determine the best method to fabricate complete dentures. The objective of this in vitro investigation was to assess and contrast the color stability and dimensional accuracy of denture base resins that were 3D printed and CAD-CAM milled, both before and after aging by thermocycling using digital surface matching technology and a benchtop laser scanner without using a spray, to optimize adaptation of the denture base and cast to minimize any imperfections and to evaluate the impact of the denture cleansing solution on the stability of color.

**Methods:**

Evaluation of the dimensional accuracy (*n* = 27) was completed on a sectional maxillary stone cast using a digital 3D-surface matching software before and after 5000 thermocycles. A spectrophotometer was used to measure the color change (△E00) of all disc specimens (*N* = 54) before and after 500 thermocycles and immersion in denture cleansing solution for 30 cycles (3 min each) daily for 6 days. The Kruskal Wallis test, Dunn’s post hoc test, Tukey’s test with Bonferroni adjustment, one sample t test and independent t test were used to statistically analyze the data (α < 0.05).

**Results:**

Thermocycling decreased the dimensional accuracy of the heat polymerized group at all 5 locations and the 3D-printed group at locations 1, 3 and 5 (*P* > .05), while it had no significant difference on the CAD-CAM milled group at all locations (*P* < .05). The color change (△E_00_) was lowest in the CAD-CAM milled group, moderate in the heat-polymerized group and highest in the 3D-printed group. After immersion in denture cleanser, the color change (△E_00_) was significantly higher in the 3 groups compared with after thermocycling (*P* > .001).

**Conclusions:**

CAD-CAM milled resins had the highest dimensional accuracy and the best color stability, conventional heat polymerized acrylic resins showed moderate change in dimensional accuracy and color stability, while the 3D- printed resin had the lowest dimensional accuracy and color stability after aging by thermocycling.

## Background

Rehabilitation with complete dentures (CDs) is the treatment of choice for completely edentulous patients who have limits due to their systemic, oral health, or financial situation [1]. The most crucial element for successful retention in removable CDs is the fit between the oral tissues and the denture base’s intaglio surface [2, 3]. Therefore, one of the goals during the process of CD fabrication is to achieve maximal tissue fit [4, 5].

Numerous studies about manufacturing techniques have been conducted to investigate the polymerization shrinkage degree [6, 7]. Before the implementation of CAD-CAM technology in removable prosthodontics, the acrylic resin’s polymerization shrinkage hindered the proper alignment between the denture base and denture-bearing tissues [8]. The polymerization shrinkage was attributed to the vaporization of residual monomers [9], inadequate surface roughness [10, 11], and porosity [12, 13].

Numerous dental prostheses are being produced using CAD-CAM technology, because it offers promising properties and excellent advantages of enhancing efficiency and productivity, time saving, and improving accuracy [7, 14]. The CAD-CAM technology for CD fabrication is being further divided into two most often used digital approaches; the subtractive milling process and the rapid prototyping system, commonly known as 3D printing or additive manufacturing [15–17].

Manufacturers of CAD-CAM pre-polymerized PMMA blocks claim their products have superior mechanical qualities over traditional denture base resins [18]. These materials have superior optical properties, color stability, and reduction of residual monomer release [9] due to their polymerization at high temperature-pressure circumstances [19–22]. In contrast to the affordable cost of benchtop 3D-printers, CAD-CAM milling machines are much expensive and this limits its use to the clinicians or dental laboratories whom can afford its cost [23]. Within the scope of literature, earlier studies have used numerous techniques and materials in an attempt to identify the optimal technique for achieving superior denture adaptation [24–26]. In order to assess the level of accuracy of any approach, it is necessary to evaluate both accuracy and precision [27].

According to the ISO standard, accuracy is how closely a measurement aligns with the correct value, while precision refers to the consistency of repeated measurements under constant circumstances [28]. In this instance, accuracy refers to the difference between the CDs’ intaglio surface made using the stone model and precision refers to how consistently the same intaglio surface is reproduced in each manufacturing technique [27]. Accelerated aging is a common testing method of materials which involves putting them under extreme circumstances that mimic the way they naturally age. The purpose of this is to mimic the effects of extended exposure to the environment by means of an accelerated weathering process [29]. Various thermocycling protocols have been proposed, but no universally standardized protocol exists [30].

A material’s color may change with age or damage. Thus, a restorative material’s stain resistance is vital [28, 31]. Both internal and external variables may cause acrylic resin to discolor [32–34]. Water sorption can happen due to extreme heat or low pressure during polymerization [13], porosity [12], residual monomers [9, 35], or poor surface properties of the resin [10, 11, 36], as well as intrinsic causes like changes to the matrix [37, 38] or extrinsic factors like absorption and adsorption [39–41].

Staining evaluation employs two primary methods: visual assessment and instrumental analysis [42, 43]. The objective methodology of color measurement surpasses visual interpretation in terms of accuracy since it greatly minimizes the inaccuracies associated with the visual method [44]. Additionally, the instrumental method eliminates the potential for subjective assessment [45]. Colorimeters and spectrophotometers are often used devices for assessing alterations in color of dental materials [44–46].

Regarding the dimensional accuracy and color stability of CAD-CAM milled and 3D-printed denture base resins, there is a lack of available data in the literature. Therefore, the research aims to evaluate and compare the dimensional accuracy and color stability of denture base resins produced by CAD-CAM milling and 3D printing methods, in comparison to the conventional heat-polymerized PMMA. Furthermore, the impact of thermal cycling on these properties, as well as the effect of a denture cleanser on color stability, will be examined.

The null hypothesis is that no difference in the dimensional accuracy and color stability exists between the conventional heat-polymerized PMMA, CAD-CAM milled and 3D-printed denture base resins prior to and following thermocycling or immersion in denture cleanser solution for color stability.

## Methods

Institutional review board permission (IORG:0008839, approval no. 0539 − 11/2022) was granted for this research by Alexandria University’s Faculty of Dentistry. Before and after thermocycling, the dimensional accuracy and color stability of PMMA-based polymers utilized as denture bases produced by CAD-CAM milling and 3D printing were assessed, and the color stability was also assessed following immersion in a denture cleanser. The sample size (*N* = 81) was estimated assuming an alpha error of 5% and 80% power of study [20, 24]. A software program (G*power 3.0.10; Heinrich Heine University Düsseldorf) was utilized to calculate the sample size using the Rosner method [47].

### Dimensional accuracy

**Fig. 1:**
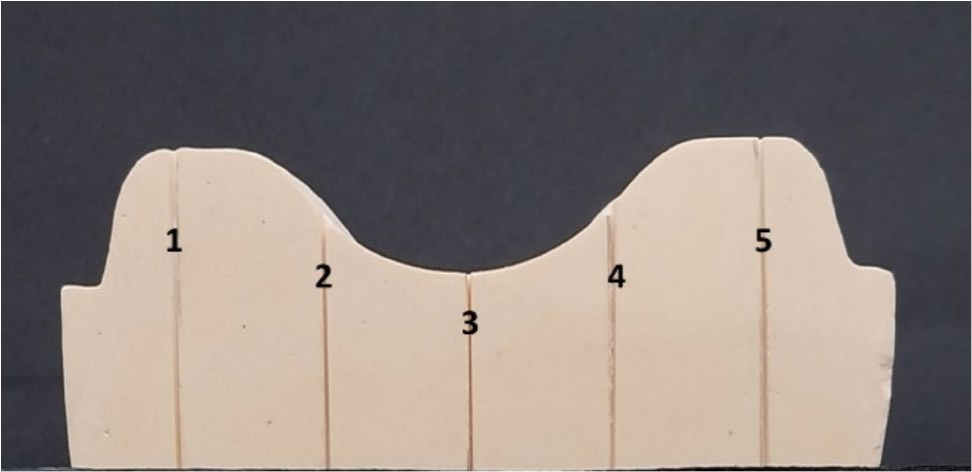
Five fine parallel lines on the stone cast

For the dimensional accuracy test, 27 specimens were allocated into 3 groups (9 specimens each). A sectional maxillary stone cast, which represents the dimensions of the edentulous ridge, was used. Five fine parallel lines were carved on the posterior aspect of the cast to be used as a reference for linear measurements of the reference points [48] for measuring dimensional accuracy (Fig. 1). A silicone mold was fabricated for the stone cast. A mix of pure extra hard type IV dental stone (Elite Master; Zhermack) was made with a constant powder: water ratio of 4:1 (100 gm powder/21 ml water) following instructions by the manufacturer and was maintained for all casts. The mix was poured into the silicone mold. This procedure was repeated to fabricate 10 identical standardized sectional stone casts.

**Fig. 2:**
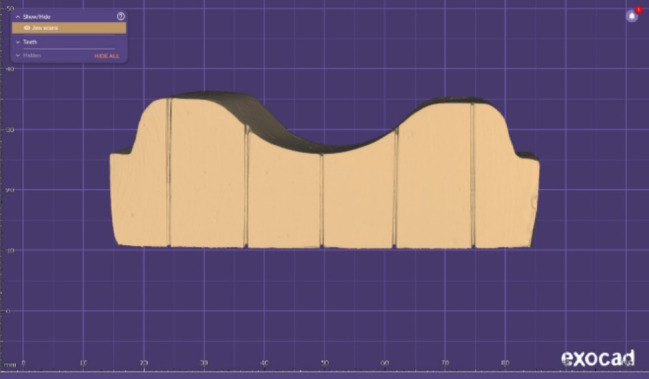
Scanned sectional stone cast

Scanning of one of the sectional master stone casts was carried out using a benchtop 3D scanner (T710; Medit). A file in the standard tessellation language (STL) format was created from the laser-scanned master stone cast (Fig. 2).

**Fig. 3:**
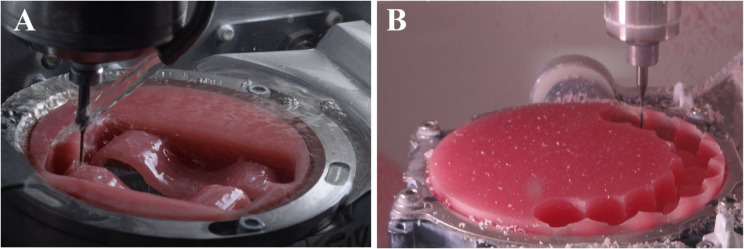
CAD-CAM milling. **(A)** Sectional denture base. **(B)** Disc specimens

A sectional complete denture base [48, 49] was created utilizing CAD software (DentalCAD; Exocad). In the present study, denture bases with a thickness of 2–3 mm were used [5, 25, 50]. This design has been utilized for direct fabrication of CAD-CAM milled denture bases (*n* = 9) (M-PM; Merz Dental) (Fig. 3) which were wet milled with a computer numerically controlled (CNC) milling machine (ED5X; EMAR Inc.).

**Fig. 4:**
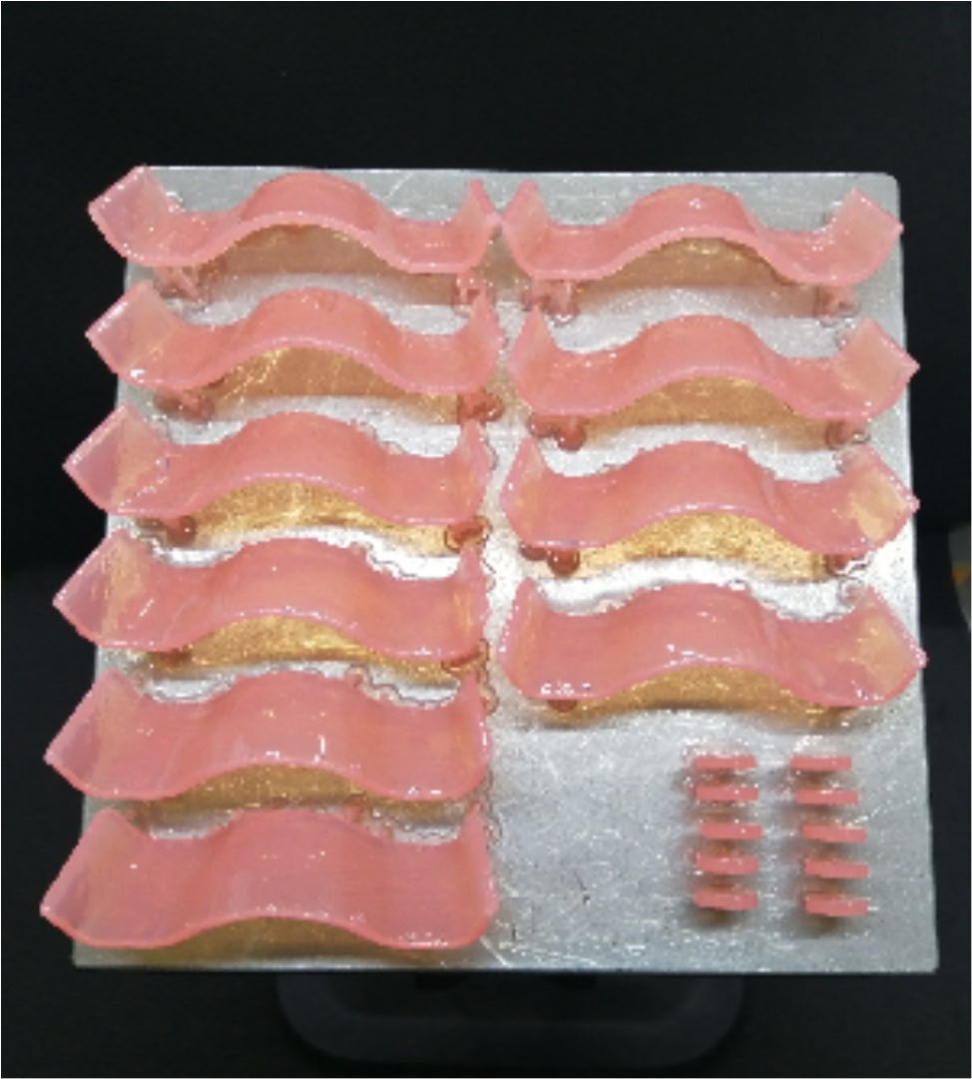
3D-printed specimens attached to the printer platform

**Fig. 5:**
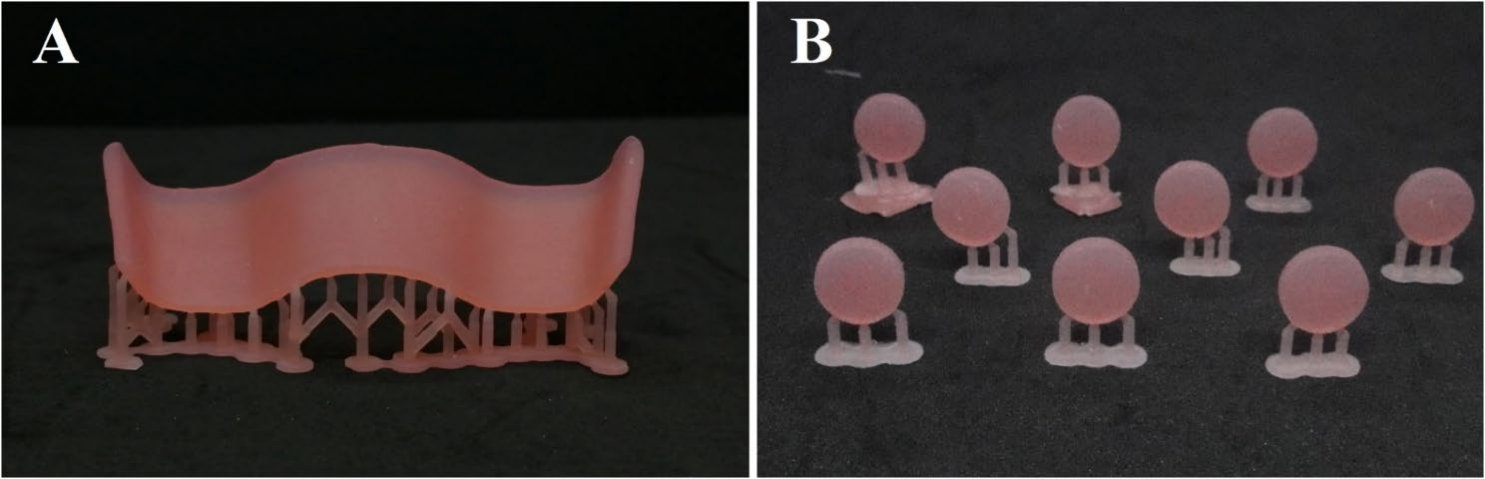
Detached 3D-printed specimens from printer platform. **A**. Sectional denture bases. **B**. Disc specimens

For the 3D-printed group, the STL file of the sectional denture base was loaded into the printer’s software program (PreForm; Formlabs). The 9 sectional denture bases were all positioned in an oblique direction toward the platform for printing [51]. Touch points measuring 0.4 mm [51] were automatically used to construct the supports. The software program’s presets were used to pick the denture base printing profile, which has a printing resolution of 50 μm [51]. The 3D printer (Form 2; Formlabs) was chosen for printing specimens using a biocompatible photopolymer resin (Denture Base OP; Formlabs) (Fig. 4). After printing, the specimens were removed from the platform of the printer (Fig. 5) and cleaned in 99% isopropyl alcohol for 10 min [51]. An ultrasonic cleaner device (Stammopur RD5; Dr. H. Stamm GmbH) was used for washing the specimens for 1 min according to manufacturer’s instructions. A UV post-polymerization machine (Form cure; Formlabs) was used to polymerize the specimens for 15 min in a glycerin solution [24].

For the heat polymerized group, wax sectional denture bases (*n* = 9) were milled, then flasked using the 9 duplicate identical casts, washed and packed with heat polymerized PMMA (SR Triplex Hot; Ivoclar Vivadent) in a conventional long curing cycle (74 °C for 8 h followed by 100 °C for 1 h) [20]. Subsequently, the flasks were left for cooling on the bench at ambient temperature for a duration of 30 min.

**Fig. 6:**
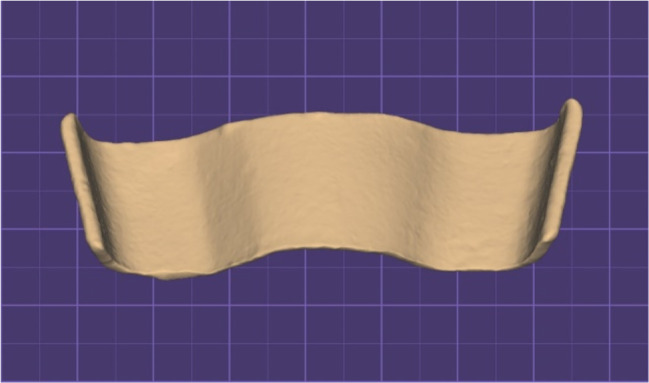
Scan of intaglio surface denture base

**Fig. 7:**
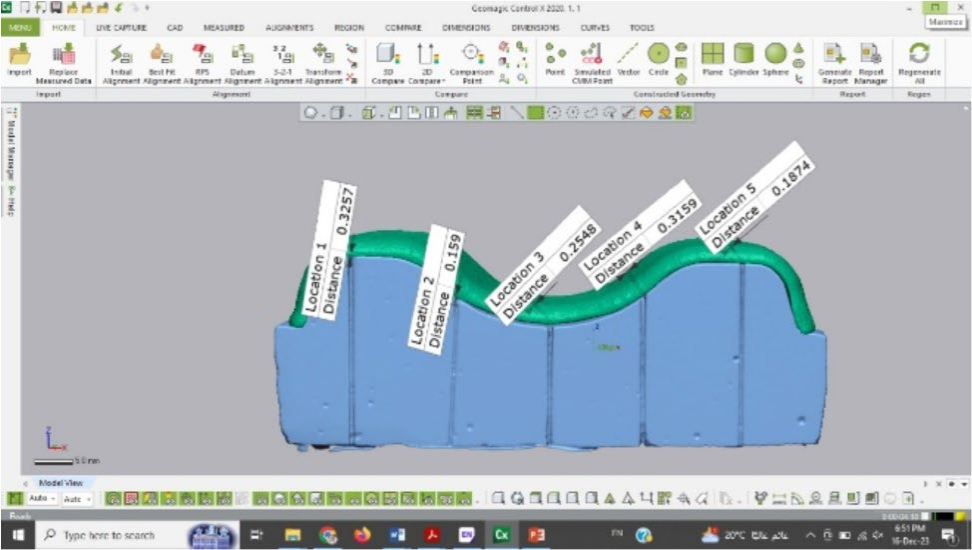
Denture base aligned to sectional master stone cast

The intaglio surfaces of each denture base were scanned in order to assess the dimensional changes in the 3 groups using the same previously used optical 3D scanner (T710; Medit) without using a scanning spray and saved as STL files (Fig. 6). Each specimen was evaluated by 2 methods: linear measurement of dimensional change at 5 reference points and 3D measurements. For the linear measurement of dimensional changes, the STL file of each sectional denture base was aligned to the STL file of the reference sectional master cast. The distance between the 5 reference points on the file of the reference sectional master cast and the same points on the files of all sectional denture bases were measured using a software program (Geomagic Control X, 3D Systems) (Fig. 7). The 5 readings named as locations 1, 2, 3, 4, and 5 were recorded for the same specimen before and after thermocycling and then compared with each other. Only one examiner scanned all intaglio surfaces. Readings at distances 1 and 5 represented the dimensional accuracy at the crest of the ridge, distance 3 represented the midline of the palate, while distances 2 and 4 represented the acrylic major connector in between them both [52].

### Thermal cycling

To study the thermocycling effect on dimensional accuracy, 27 specimens were stored in an incubator (Binder BD-53, Binder GmbH) [1] at 37 ± 2 °C distilled water for 24 h, following the ISO/TR 11,405: 1994 [53]. A thermocycling machine (Huber 1100, SD Mechatronik) [44] was used to subject the specimens to 5000 cycles, with a dwell time of 30 s and a transfer time of 20 s to simulate 5 years of denture use [54, 55].

### Superimposition

For the 3D measurements of dimensional changes, the STL file for each sectional denture base before thermocycling and that of the same base after thermocycling were superimposed using the software program (Geomagic Control X, 3D Systems). Evaluation of dimensional accuracy of each sample was completed before and after thermocycling for the 3 groups in the study.

### Color stability

The color stability test specimens, consisting of 54 discs measuring Ø10 × 2 mm [46], were divided into three groups: CAD-CAM milled (*N* = 18), 3D-printed (*N* = 18), and heat-polymerized PMMA group (*N* = 18).

After completing the processing steps of all disc specimens (*N* = 54), they were polished on both sides using a lathe machine with a rag wheel. The polishing process included using a slurry of pumice (Steribim super; BEGO GmbH) previously mixed with water for 90 s at 1500 rpm, then applying polishing paste (Universal Polishing Paste; Ivoclar AG) for an additional 90 s [51]. A skilled operator polished the specimens to ensure consistent force was applied. Following the polishing process, all specimens underwent quality control assessment using a digital caliper (Digimatic AOS, Mituyoto) to reconfirm their dimensions [56]. After being sanitized for five minutes with an 80% ethanol solution, the disc specimens were manually dried using sterile gauze [57].

In order to investigate the impact of thermocycling on color stability, a total of 27 samples (9 per group) underwent 500 cycles of thermocycling using (Huber 1100, SD Mechatronik) a thermocycling equipment [44]. This was done to imitate 6 months of denture use [51]. A spectrophotometer (Agilent Cary 5000; Agilent Technologies) was used to measure color change of every specimen following ISO 9001 [58] Color measurements were obtained using the D65 standard illuminant to replicate daylight conditions and a 10-degree viewing angle [42]. Each disc specimen’s color laboratory values were measured before and after thermocycling. A single proficient operator recorded the measurements.

To examine the effects of denture cleanser on color stability, a total of 27 samples were allocated to 3 groups (9 per group) and submerged in a denture cleanser solution (Corega Tablet, Block Drug Co.). Table 1 displays the composition of the denture cleanser used in the study [59, 60]. To simulate 180 days of complete denture usage, a total of 30 submersion cycles, each lasting 3 min, were conducted on a daily basis for 6 days [61]. The dentures were submerged in warm water at a temperature of 40℃ and a denture cleansing tablet was added [45]. Every immersion cycle was conducted using a newly manufactured denture cleansing solution. Specimens were cleaned by submerging them in freshly prepared distilled water that was left at room temperature [62]. The analysis, processes, transfers, and renewals were performed at a constant room temperature of 23 ± 2 °C. One operator fulfilled these tasks by following ISO 20795-1:2013 requirements [63].

**Table 1:** The manufacturer and composition of corega tablets

Material’s trade name	Main composition	Fabrication technique
Correga Tablet (Block Drug Company, Inc., NJ, USA)	Sodium bicarbonate, citric acid, sodium perborate monohydrate, potassium peroxymonosulfate, sodium benzoate, sodium lauryl sulfoacetate, peppermint flavor, subtilisin	30 submersion cycles (3 min each) were performed each day for 6 days to imitate 180 days of prosthesis submersion by the patient

The CIEDE 2000 color difference formula, developed by the International Commission of Illumination (CIE), was used to calculate color change values (ΔE). The ΔE00 value between the two samples was computed using the following formula: [19]

$$\:{\varDelta\:E}_{00}={\left[{\left(\frac{\varDelta\:{\varvec{L}}^{{\prime\:}}}{{\varvec{K}}_{\varvec{L}}{\varvec{S}}_{\varvec{L}}}\right)}^{2}+{\left(\frac{\varDelta\:{\varvec{C}}^{{\prime\:}}}{{\varvec{K}}_{\varvec{C}}{\varvec{S}}_{\varvec{C}}}\right)}^{2}+{\left(\frac{\varDelta\:{\varvec{H}}^{{\prime\:}}}{{\varvec{K}}_{\varvec{H}}{\varvec{S}}_{\varvec{H}}}\right)}^{2}+{\varvec{R}}_{\varvec{T}}\left(\frac{\varDelta\:{\varvec{C}}^{{\prime\:}}}{{\varvec{K}}_{\varvec{C}}{\varvec{S}}_{\varvec{C}}}\right)\left(\frac{\varDelta\:{\varvec{H}}^{{\prime\:}}}{{\varvec{K}}_{\varvec{H}}{\varvec{S}}_{\varvec{H}}}\right)\right]}^{\frac{1}{2}}$$

For the specimens, the background was selected with the parameters (L*=10.38, a*=5.87, b*=0.90). Ĺ, Ć, and Ĥ stand for lightness, chroma, and hue, respectively, while RT and S indicate the rotation and weighing functions, respectively. The correction terms for the experimental conditions, namely kL, kC, and kH, were set to a value of 1 [64].

The PMMA disc specimens were compared using a perceptibility and acceptability threshold test to assess their color change after aging, both among themselves and in comparison to the color change considered clinically acceptable. The term “perceptibility” refers to the degree of color change that can be seen by the human eye, while “acceptability” pertains to the color change that is considered both clinically and aesthetically satisfactory [65, 66]. The National Bureau of Standards (NBS) measured the color change of acrylic resin base materials concerning the clinical environment. They used the formula NBS units = ΔE × 0.92 (Table 2) to quantify the color variations [59].

**Table 2:** The National Bureau of Standards (NBS) units of color difference

Marks of color difference	NBS units
Trace	0.0–0.5
Noticeable	0.5–1.5
Slight	1.5 – 3
Appreciable	3 – 6
Much	6 – 12
Very Much	> 12

The statistical analysis was conducted using IBM SPSS Statistics for Windows, v23 (IBM Corp.), a statistical software package. Q-Q plots and the Shapiro-Wilk test were used to verify normality. Dimensional accuracy (superimposition and alignment) was not normally distributed while color parameters were normally distributed. Dimensional accuracy was compared between groups using the Kruskal Wallis test followed by Dunn’s post hoc test with Bonferroni correction. To compare color parameters between the groups, one way ANOVA followed by Tukey’s test with Bonferroni adjustment were used. To compare color change between thermocycling and denture cleansers, independent t test was performed. To assess the difference in color change in each group in relation to perceptibility threshold and acceptability threshold one sample t test was performed (α = 0.05 for all tests).

## Results

**Fig. 8:**
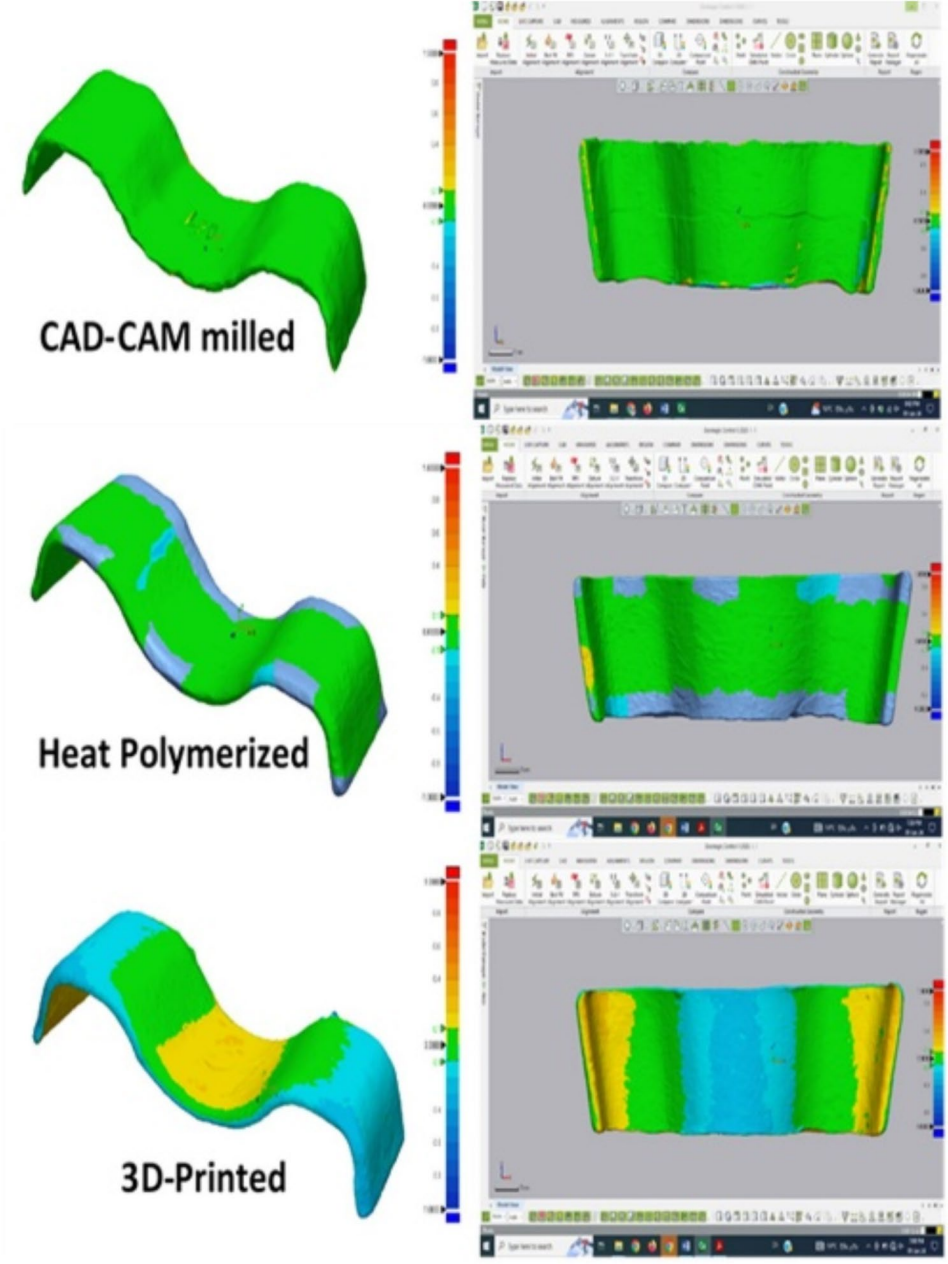
Color map showing superimposition of denture bases

The color maps of the 3 groups were obtained by applying superimposition of each denture base to itself before and after thermocycling (Fig. 8). The CAD-CAM milled technique demonstrated the highest dimensional accuracy and optimal distribution of adaption in the color map, while the 3D-printed group showed the lowest dimensional accuracy and least uniform distribution of adaptation in the color map and the conventional heat polymerized PMMA lies in between them both. Areas that are yellow, red or blue demonstrate a change in the dimensions of this denture base that occurred after thermocycling. An optimal denture base would exhibit a color map consisting only of green, indicating a measurement value of 0. If the denture base were to fit perfectly into the cast, this value would indicate that there was no processing deformation.

Table 3 shows the differences in dimensional accuracy alignment and spaces at different locations before and after thermocycling using the Wilcoxson Sign Rank test. By applying alignment of the denture base on the reference study cast, it was found that thermocycling decreased the dimensional accuracy of the heat polymerized group at all 5 locations and the 3D-printed group at locations 1, 3 and 5 (*P* > .05), while it had no significant difference on the 3D-printed group at both locations 2 and 4 and on the CAD-CAM milled group at all the 5 locations (*P* < .05).

**Table 3:** Comparison of spaces in mm at each location before and after thermocycling among the study groups

		Heat polymerized		CAD-CAM milled		3D-printed	
		(*n* = 9)		(*n* = 9)		(*n* = 9)	
		Before	After	Before	After	Before	After
Location 1	Mean ± SD	0.23 ± 0.13	0.40 ± 0.14	0.07 ± 0.01	0.07 ± 0.01	0.24 ± 0.12	0.54 ± 0.34
	Median (IQR)	0.19 (0.25)	0.41 (0.14)	0.07 (0.02)	0.07 (0.02)	0.21 (0.17)	0.52 (0.41)
	Min - Max	0.10–0.44	0.22–0.71	0.06–0.09	0.06–0.09	0.13–0.48	0.18–1.31
	Wilcoxon Test	2.66		1		2.66	
	(*p* value)	(0.008*)		-0.317		(0.008*)	
Location 2	Mean ± SD	0.43 ± 0.36	0.85 ± 0.44	0.08 ± 0.01	0.08 ± 0.01	0.51 ± 0.25	0.75 ± 0.65
	Median (IQR)	0.37 (0.52)	0.72 (0.74)	0.08 (0.02)	0.08 (0.0)	0.48 (0.41)	0.58 (1.33)
	Min - Max	0.08–1.23	0.25–1.56	0.07–0.10	0.07–0.10	0.17–0.95	0.07–1.64
	Wilcoxon Test	2.66		1.401		1.01	
	(*p* value)	(0.008*)		-0.132		-0.314	
Location 3	Mean ± SD	0.55 ± 0.32	0.99 ± 0.37	0.08 ± 0.01	0.08 ± 0.01	0.85 ± 0.38	1.36 ± 0.35
	Median (IQR)	0.52 (0.43)	0.93 (0.54)	0.09 (0.01)	0.09 (0.01)	0.80 (0.48)	1.42 (0.46)
	Min - Max	0.11–1.13	0.53–1.61	0.07–0.09	0.07–0.09	0.12–1.43	0.63–1.77
	Wilcoxon Test	2.66		1.414		2.66	
	(*p* value)	(0.008*)		-0.157		(0.008*)	
Location 4	Mean ± SD	0.54 ± 0.34	1.03 ± 0.37	0.07 ± 0.02	0.07 ± 0.03	0.54 ± 0.28	0.78 ± 0.29
	Median (IQR)	0.47 (0.42)	0.92 (0.57)	0.07 (0.02)	0.07 (0.02)	0.57 (0.55)	0.90 (0.19)
	Min - Max	0.11–1.26	0.54–1.72	0.05–0.09	0.05–0.09	0.16–0.93	0.08–1.10
	Wilcoxon Test	2.66		1.732		1.59	
	(*p* value)	(0.008*)		-0.083		-0.11	
Location 5	Mean ± SD	0.23 ± 0.08	0.35 ± 0.09	0.08 ± 0.02	0.08 ± 0.02	0.42 ± 0.17	0.81 ± 0.11
	Median (IQR)	0.23 (0.13)	0.39 (0.16)	0.08 (0.03)	0.08 (0.03)	0.38 (0.32)	0.79 (0.21)
	Min - Max	0.10–0.37	0.20–0.44	0.06–0.10	0.06–0.10	0.22–0.69	0.69–0.98
	Wilcoxon Test	2.66		1.7		2.66	
	(*p* value)	(0.008*)		-0.081		(0.008*)	

*Statistically significant difference at *P* value < 0.05

**Fig. 9:**
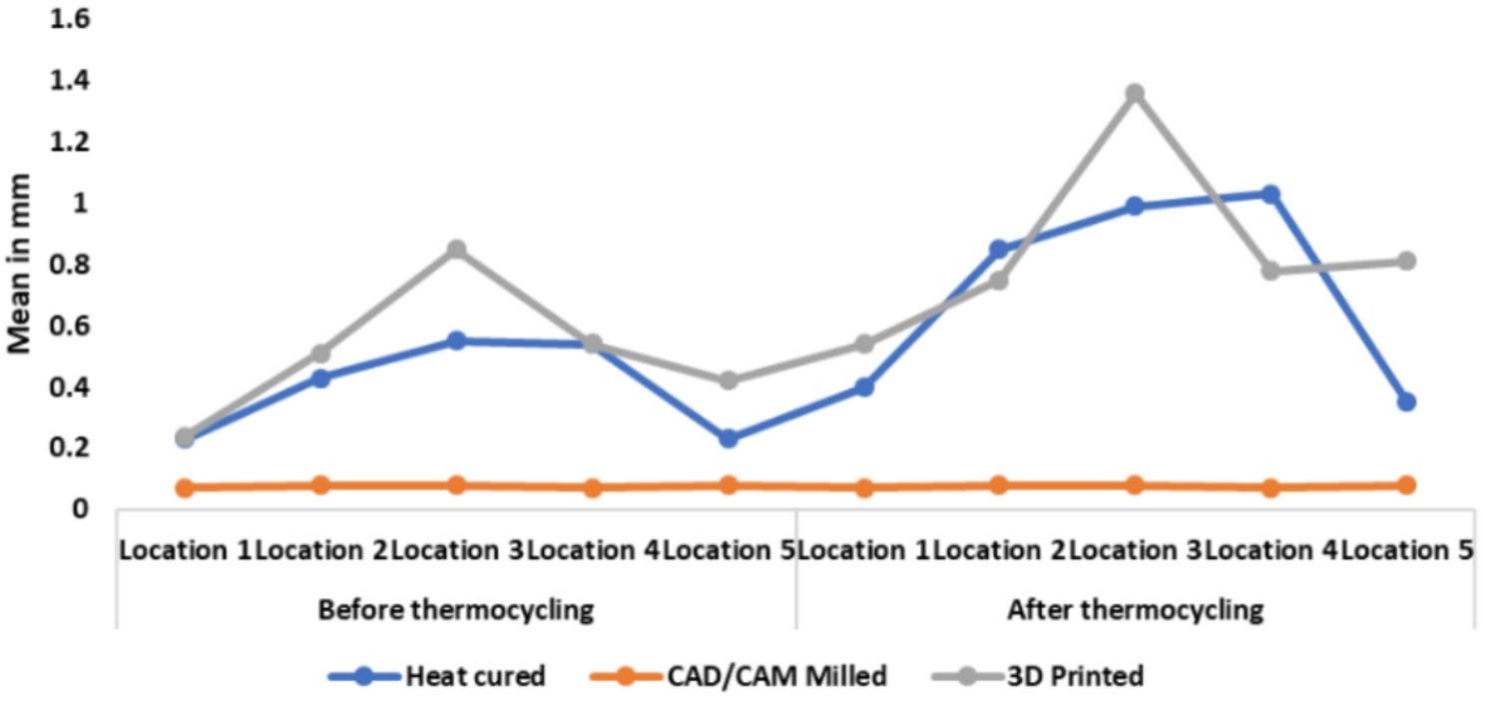
Line graphs demonstrating the observations for each of the 3 processing techniques within each location before and after thermocycling

Tables 4 and 5 show a comparison of dimensional accuracy at each location between groups using the Kruskal Wallis test, followed by Dunn’s post hoc test with Bonferroni correction. Line graphs were constructed to demonstrate the observations for each processing technique at each location before and after thermocycling (Fig. 9). The dimensional accuracy of the CAD-CAM milled group at all 5 locations was greater compared to the heat polymerized PMMA group and the 3D-printed group whether before or after thermocycling (*P* > .01). However, at location 4, it was found that after thermocycling, there was no significant difference between the dimensional accuracy of the CAD-CAM milled group and the 3D-printed group (*P* < .05). When measuring dimensional accuracy at locations 1, 2 and 3, no significant difference existed between the heat polymerized PMMA group and the 3D-printed group either before or after thermocycling (*P* < .05). The heat polymerized PMMA group displayed greater dimensional accuracy compared to the 3D-printed group after thermocycling at locations 4 and 5 (*P* > .05).

**Table 4:** Comparison of spaces in mm at each location before and after thermocycling among the study groups after denture cleanser

			Heat polymerized	CAD/CAM milled	3D-printed	Kruskal wallis test
			(*n* = 9)	(*n* = 9)	(*n* = 9)	*(p* value)
Before thermocycling	Location 1	Mean ± SD	0.23 ± 0.13	0.07 ± 0.01	0.24 ± 0.12	17.617
		Median (IQR)	0.19 (0.25)	0.07 (0.02)	0.21 (0.17)	(< 0.001*)
		Min - Max	0.10–0.44	0.06–0.09	0.13–0.48	
	Location 2	Mean ± SD	0.43 ± 0.36	0.08 ± 0.01	0.51 ± 0.25	15.977
		Median (IQR)	0.37 (0.52)	0.08 (0.02)	0.48 (0.41)	(< 0.001*)
		Min - Max	0.08–1.23	0.07–0.10	0.17–0.95	
	Location 3	Mean ± SD	0.55 ± 0.32	0.08 ± 0.01	0.85 ± 0.38	19.143
		Median (IQR)	0.52 (0.43)	0.09 (0.01)	0.80 (0.48)	(< 0.001*)
		Min - Max	0.11–1.13	0.07–0.09	0.12–1.43	
	Location 4	Mean ± SD	0.54 ± 0.34	0.07 ± 0.02	0.54 ± 0.28	17.384
		Median (IQR)	0.47 (0.42)	0.07 (0.02)	0.57 (0.55)	(< 0.001*)
		Min - Max	0.11–1.26	0.05–0.09	0.16–0.93	
	Location 5	Mean ± SD	0.23 ± 0.08	0.08 ± 0.02	0.42 ± 0.17	20.33
		Median (IQR)	0.23 (0.13)	0.08 (0.03)	0.38 (0.32)	(< 0.001*)
		Min - Max	0.10–0.37	0.06–0.10	0.22–0.69	
After thermocycling	Location 1	Mean ± SD	0.40 ± 0.14	0.07 ± 0.01	0.54 ± 0.34	17.829
		Median (IQR)	0.41 (0.14)	0.07 (0.02)	0.52 (0.41)	(< 0.001*)
		Min - Max	0.22–0.71	0.06–0.09	0.18–1.31	
	Location 2	Mean ± SD	0.85 ± 0.44	0.08 ± 0.01	0.75 ± 0.65	13.39
		Median (IQR)	0.72 (0.74)	0.08 (0.0)	0.58 (1.33)	(0.001*)
		Min - Max	0.25–1.56	0.07–0.10	0.07–1.64	
	Location 3	Mean ± SD	0.99 ± 0.37	0.08 ± 0.01	1.36 ± 0.35	19.149
		Median (IQR)	0.93 (0.54)	0.09 (0.01)	1.42 (0.46)	(< 0.001*)
		Min - Max	0.53–1.61	0.07–0.09	0.63–1.77	
	Location 4	Mean ± SD	1.03 ± 0.37	0.07 ± 0.03	0.78 ± 0.29	16.696
		Median (IQR)	0.92 (0.57)	0.07 (0.02)	0.90 (0.19)	(< 0.001*)
		Min - Max	0.54–1.72	0.05–0.09	0.08–1.10	
	Location 5	Mean ± SD	0.35 ± 0.09	0.08 ± 0.02	0.81 ± 0.11	23.157
		Median (IQR)	0.39 (0.16)	0.08 (0.03)	0.79 (0.21)	(< 0.001*)
		Min - Max	0.20–0.44	0.06–0.10	0.69–0.98	

*Statistically significant difference at *P* value < 0.05

**Table 5:** Pairwise comparison of spaces at each location between the study groups before and after thermocycling

Locations	Groups	Compared to	*p* value	
			Before	After
**Location 1**	Heat polymerized	CAD/CAM Milled	0.002*	0.003*
		3D-printed	1.00	1.00
	CAD/CAM Milled	3D-printed	< 0.001*	< 0.001*
**Location 2**	Heat polymerized	CAD/CAM Milled	0.009*	0.002*
		3D-printed	1.00	1.00
	CAD/CAM Milled	3D-printed	< 0.001*	0.016*
**Location 3**	Heat polymerized	CAD/CAM Milled	0.010*	0.010*
		3D-printed	0.544	0.544
	CAD/CAM Milled	3D-printed	< 0.001*	< 0.001*
**Location 4**	Heat polymerized	CAD/CAM Milled	0.001*	< 0.001*
		3D-printed	1.00	0.006*
	CAD/CAM Milled	3D-printed	0.001*	1.00
**Location 5**	Heat polymerized	CAD/CAM Milled	0.018*	0.048*
		3D-printed	0.255	0.048*
	CAD/CAM Milled	3D-printed	< 0.001*	< 0.001*

*Statistically significant difference at *P* value < 0.05

The ranking of all 5 locations according to their reproducibility (IQR) and accuracy (median values) are listed in Tables 6 and 7. As the result value approaches 0, it indicates that the technique was performed with a higher level of accuracy and reproducibility [25]. The median and IQR range values for the CAD-CAM milled group were consistently located around zero at all five locations, both before and after thermocycling. Hence, the milling technique showed optimal accuracy as well as reproducibility compared to the other processing techniques that were tested. The 3D printing technique produced the highest median results at 3 out of 5 locations, whether before or after thermocycling, and therefore, the lowest accuracy, while the median results of the heat polymerized group lied in between them both. The 3D printing technique produced the highest values of interquartile range (IQR) at 3 out of 5 locations and therefore the lowest reproducibility whether before or after thermocycling. The heat polymerizing technique showed the highest values of IQR at 2 out of 5 locations whether before or after thermocycling and therefore they possess moderate reproducibility.

**Table 6:** Accuracy of processing ranking based on location before and after thermocycling

	Before thermocycling		After thermocycling	
	Rank of accuracy: proximity to zero (Median)		Rank of accuracy: proximity to zero (Median)	
**Location 1**	1. CAD-CAM Milled	0.07	1. CAD-CAM Milled	0.07
	2. Heat Polymerized	0.19	2. Heat Polymerized	0.41
	3. 3-D Printed	0.21	3. 3-D Printed	0.52
**Location 2**	1. CAD-CAM Milled	0.08	1. CAD-CAM Milled	0.08
	2. Heat Polymerized	0.37	2. 3-D Printed	0.58
	3. 3-D Printed	0.48	3. Heat Polymerized	0.72
**Location 3**	1. CAD-CAM Milled	0.09	1. CAD-CAM Milled	0.09
	2. Heat Polymerized	0.52	2. Heat Polymerized	0.93
	3. 3-D Printed	0.80	3. 3-D Printed	1.42
**Location 4**	1. CAD-CAM Milled	0.07	1. CAD-CAM Milled	0.07
	2. Heat Polymerized	0.47	2. 3-D Printed	0.90
	3. 3-D Printed	0.57	3. Heat Polymerized	0.92
**Location 5**	1. CAD-CAM Milled	0.08	1. CAD-CAM Milled	0.08
	2. Heat Polymerized	0.23	2. Heat Polymerized	0.39
	3. 3-D Printed	0.38	3. 3-D Printed	0.79

**Table 7:** Reproducibility of processing ranking based on location before and after thermocycling

	Before thermocycling		After thermocycling	
	Rank of reproducibility: proximity to zero (IQR)		Rank of reproducibility: proximity to zero (IQR)	
**Location 1**	1. CAD-CAM Milled	0.02	1. CAD-CAM Milled	0.02
	2. 3D-Printed	0.17	2. Heat Polymerized	0.14
	3. Heat Polymerized	0.25	3. 3-D Printed	0.41
**Location 2**	1. CAD-CAM Milled	0.02	1. CAD-CAM Milled	0.0
	2. 3D-Printed	0.41	2. Heat Polymerized	0.74
	3. Heat Polymerized	0.52	3. 3-D Printed	1.33
**Location 3**	1. CAD-CAM Milled	0.01	1. CAD-CAM Milled	0.09
	2. Heat Polymerized	0.43	2. 3D-Printed	0.46
	3. 3D-Printed	0.48	3. Heat Polymerized	0.54
**Location 4**	1. CAD-CAM Milled	0.02	4. CAD-CAM Milled	0.02
	2. Heat Polymerized	0.42	5. 3-D Printed	0.19
	3. 3D-Printed	0.55	6. Heat Polymerized	0.57
**Location 5**	1. CAD-CAM Milled	0.03	4. CAD-CAM Milled	0.03
	2. Heat Polymerized	0.13	5. Heat Polymerized	0.16
	3. 3D-Printed	0.32	6. 3-D Printed	0.21

Table 8 shows the color change (△E_00_) among the study groups after thermocycling. The CAD-CAM milled group showed the lowest change in color and, therefore, the highest color stability (△E_00_ = 0.911 ± 0.075), followed by conventional heat-polymerized group (△E_00_ = 1.366 ± 0.110), while the 3D-printed group showed the greatest color change (△E_00_ = 1.957 ± 0.129), with a significant difference between the 3 groups (*P* < .001).

**Table 8:** Comparison of color change ∆E_00_ among the study groups after thermocycling

		Heat polymerized (*n* = 9)	CAD-CAM milled (*n* = 9)	3D-printed (*n* = 9)
∆E_00_	Mean ± SD	1.366 ± 0.110	0.911 ± 0.075	1.957 ± 0.129
	Median (IQR)	1.395 (0.211)	0.922 (0.121)	2.002 (0.238)
	Min - Max	1.199–1.481	0.789–0.990	1.761–2.099
	F test (*p* value)	214.701 (< 0.001*)		
	Pairwise comparison	*p*_1_ < 0.001*, *p*_*2*_ < 0.001*, *p*_*3*_ *<* 0.001*		

*Statistically significant difference at *P* < .05, *p*_1_: comparison between heat polymerized and milled, *p*_2_: comparison between heat polymerized and 3D-printed, *p*_3_: comparison between milled and 3D-printed

Table 9 shows the color change (△E_00_) among the study groups after immersion in a denture cleanser. The CAD-CAM milled group showed the lowest color change (△E_00_ = 2.012 ± 0.353), followed by the conventional heat polymerized group (△E_00_ = 2.991 ± 0.244), then the 3D-printed group showed the greatest color change (△E_00_ = 3.185 ± 0.389), with a significant difference between the milled group compared to heat polymerized and the 3D-printed group (*P* < .001).

**Table 9:** Comparison of color change ∆E_00_ among the study groups after denture cleanser

		Heat polymerized (*n* = 9)	CAD-CAM milled (*n* = 9)	3D-printed (*n* = 9)
∆E_00_	Mean ± SD	2.991 ± 0.244	2.012 ± 0.353	3.185 ± 0.389
	Median (IQR)	2.965 (0.373)	2.025 (0.510)	3.215 (0.520)
	Min - Max	2.700–3.500	1.400–2.650	2.550–3.920
	F test (*p* value)	35.320 (< 0.001*)		
	Pairwise comparison	*p*_1_ < 0.001*, *p*_*2*_ = 0.409, *p*_*3*_ *<* 0.001*		

*Statistically significant difference at *P* < .05, *p*_1_: comparison between heat polymerized and milled, *p*_2_: comparison between heat polymerized and 3D-printed, *p*_3_: comparison between milled and 3D-printed

Table 10 compares color change (△E_00_) between aging by thermocycling and denture cleanser. When subjected to denture cleanser, the △E_00_ of the 3 groups was greater than that of thermocycling with a significant difference (*P* > .001).

**Table 10:** Comparison of color change ∆E_00_ between thermocycling and denture cleanser among the study groups

		Heat polymerized	CAD-CAM milled	3D-printed
Thermocycling	Mean ± SD	1.366 ± 0.110	0.911 ± 0.075	1.957 ± 0.129
	Median (IQR)	1.395 (0.211)	0.922 (0.121)	2.002 (0.238)
	Min - Max	1.199–1.481	0.789–0.990	1.761–2.099
Denture cleanser	Mean ± SD	2.991 ± 0.244	2.012 ± 0.353	3.185 ± 0.389
	Median (IQR)	2.965 (0.373)	2.025 (0.510)	3.215 (0.520)
	Min - Max	2.700–3.500	1.400–2.650	2.550–3.920
Independent t test (*p* value)		18.3307 (< 0.001*)	9.179 (< 0.001*)	8.979 (< 0.001*)

*Statistically significant difference at *P* < .05

Table 11 compares the color change (△E) in relation to the perceptibility and acceptability threshold. After thermocycling, the color changes (△E) of both the 3D-printed group and heat-polymerized group were below the clinical perceptibility and acceptability threshold with a significant difference (*P* < .001), and the CAD-CAM milled group was significantly lower than the acceptability threshold (*P* < .001). However, there was no statistically significant difference in the CAD-CAM milled group regarding the perceptibility threshold (*P* = .174). After denture cleanser, the △E of the 3 groups were above the clinical perceptibility threshold with a significant difference (*P* < .001). However, △E of the CAD-CAM milled group and the 3D-printed group were far below the clinical acceptability threshold with a statistically significant difference (*P* < .001 and = 0.022 respectively), while it had no significant difference on the heat-polymerized group (*P* = .424).

**Table 11:** Comparison of ∆E in relation to perceptibility threshold and acceptability threshold

Aging process	Groups	Perceptibility threshold (∆E = 1.2)			Acceptability threshold (∆E = 2.7)		
		Mean diff	95% CI	*p* value	Mean diff	95% CI	*p* value
**After thermocycling**	**Heat polymerized**	0.322	0.237, 0.408	< 0.001*	-1.578	-1.663, -1.492	< 0.001*
	**CAD-CAM milled**	-0.042	-0.107, 0.023	0.174	-1.942	-2.007, -1.877	< 0.001*
	**3D-printed**	0.997	0.894, 1.100	< 0.001*	-0.903	-1.006, -0.800	< 0.001*
**After denture cleanser**	**Heat polymerized**	1.965	1.789, 2.142	< 0.001*	0.065	-0.111, 0.242	0.424
	**CAD-CAM milled**	1.155	0.882, 1.428	< 0.001*	-0.745	-1.018, -0.472	< 0.001*
	**3D-printed**	2.247	1.963, 2.531	< 0.001*	0.347	0.063, 0.631	0.022*

*Statistically significant difference at *P* < .05, CI: Confidence Interval

## Discussion

The study aimed to test the impact of thermocycling on dimensional accuracy and stability of color, and the influence of a denture cleanser on the stability of color of conventional, CAD-CAM milled and 3D-printed denture base resins. The null hypotheses that thermocycling has no significant effect on dimensional accuracy and color stability of milled, 3D-printed or conventional heat-polymerized denture bases and that the denture cleanser would have no effect on color stability, were rejected.

Dimensional accuracy is considered an essential factor in the success of complete denture bases [7]. Denture accuracy, retention, and stability are influenced by the polymerization shrinkage [4]. The adaptation of denture bases was assessed in earlier studies using a physical measurement of inaccuracies [3, 7, 50]. In this study, a surface matching process was applied using digital technology by a single operator and examiner using a benchtop laser scanner without the use of a spray, to optimize the adaptation of the denture base and cast, achieving a precise fit and minimize any imperfections.

The results in the present study demonstrated that thermocycling had no significant impact on the dimensional accuracy of the milled group, while it decreased the dimensional accuracy of the 3D-printed group at the crest of the ridge and midline of the palate. This may be because changes in the physical properties of 3D-printed resin materials caused by water sorption as it is being printed in successive layers [20]. In addition to incomplete bonding, large amounts of residual monomer leaking during post-polymerization processes after printing, in addition to low polymerization rate, are the reasons that 3D-printed resins are more liable to dimensional change with thermal aging [20]. Thermocycling decreased the dimensional accuracy of the heat polymerized PMMA at all 5 locations of the denture base. These results are consistent with Helal et al. [55], Goodacre et al. [25], and Lee et al. [7] The conventional heat polymerized PMMA is liable to insufficient pressure or excessive heating during exothermic polymerization reaction which can cause an excessive amount of residual monomers to leach out [9] and result in poor surface roughness [10, 11]. Temperature changes might weaken polymer chains and eventually lead to water sorption with hygroscopic expansion or porosity [12, 13]. 

It seems like the findings from the median values and the Kruskal-Wallis analysis often contradict each other. Keep in mind that the Kruskal-Wallis test does not only simulate a median value; it considers all data. Kruskal-Wallis analysis compares each measurement point with its equivalent point from the other processing approach, whereas the median offers an entire perspective of the data [25]. 

Due to this variation, the recognition of a statistically significant difference may not necessarily align with the difference between median values. An example of this can be seen at locations 1 and 5 (representing the crest of the ridge) before thermocycling, where the heat polymerized and 3D printed groups did not exhibit a substantially significant difference, but their median values seemed to be significant. The range of measurements recorded is more important than the mean or median when comparing the 3 processing techniques’ capabilities to create a denture that is both accurate and dimensionally stable [25]. Assessing the accuracy and reproducibility of every processing method is crucial to ascertain the method with the highest level of dimensional accuracy. An accurate processing technique should produce a denture base having a median dimensional accuracy that is close to zero, while a reproducible processing technique can create a denture base having the smallest interquartile range possible of dimensional accuracy measurement [25]. 

The CAD-CAM milling technique had the greatest dimensional accuracy at all 5 locations of the reference stone cast followed by the heat polymerized PMMA group then the 3D-printed group showed the least dimensional accuracy before and after thermocycling. However, the heat polymerized PMMA group showed the least accuracy at locations 2 and 4 after thermocycling. These results are consistent with Goodacre et al. [25], Steimassl et al. [8], and Helal et al. [55]There are multiple reasons for the high dimensional accuracy of the milled group. Those materials are pre-polymerized at optimal conditions under high temperature and high pressure. As a result, they possess a high rate of polymerization, minimal residual monomer, less porous, and more compact structure [19]. 

The CAD-CAM milled group was comparatively superior in accuracy to the conventional acrylic resin materials. This is due to changes in the physical properties of the resin materials, in addition to a higher probability of processing defects such as micro-porosity [12] that might occur during the curing cycle of heat polymerized PMMA group which weakens polymer chains and eventually leads to water sorption without hygroscopic expansion which leads to dimensional misfit [13]. The CAD-CAM milling technique was the best technique concerning reproducibility at all 5 locations of the denture base before and after thermocycling, followed by the heat polymerized group, then the 3D-printed group which showed the lowest reproducibility.

Dentists use a scale called the perceptibility threshold (PT) to describe the point at which half of the observers notice a color change and an acceptability threshold (AT) to describe the point at which half of the observers, under controlled settings, find the change acceptable [56]. The 50% perceptibility threshold (PT) and 50% acceptability threshold (AT) values published by CIELab are 1.2 and 2.7, respectively [65, 66]. After thermocycling or immersion in chemical cleaning agents, all three groups of denture base resins demonstrated clinically acceptable ΔE values (NBS Units 6 > and △E < 2.7) in this investigation [59]. This result is consistent with Jain et al. [62]. In contrast, it is opposite to Alfouzan et al. [44] This may be due to different immersion times in the denture cleansing solution and a different number of cycles in the thermocycling machine.

Susceptibility of acrylic resin to discoloration depends on characteristics of the denture base resins. Sorption of liquids is the main causative factor for staining of acrylic resin. Water sorption and hygroscopic expansion cause base materials used in dentures to change color in a direct proportion [13]. Additionally, any denture base resin with water-absorbing properties will also be able to take up other liquids containing substances that cause discoloration [13]. When water sorption occurs, the polymer matrix of the denture base resin stretches and cleaves the polymer chains. This facilitates the penetration of staining agents to the denture base material. When porosity occurs, it permits water sorption without hygroscopic expansion [10]. 

Upon comparing the three groups, it was seen that there was a significant change in color. However, the extent of the color variation varied depending on the method of production and the kind of material used for the denture foundation. Spectrophotometry is the predominant and precise technique for measuring color, since it provides a quantitative and unbiased assessment of the change in color of a substance [44]. 

When discs were thermocycled, the least amount of color change was seen in the CAD-CAM milled group, followed by heat polymerized group then the 3D-printed group (*P* > .001). There are multiple reasons of high color stability of milled PMMA groups.

The great color stability of milled PMMA groups may be attributed to many factors.Those materials are pre-polymerized at optimal conditions under high temperature and high-pressure. As a result, they possess high rate of polymerization, minimal residual monomer, less porous and compact structure [19]. In contrast, the 3D-printed group showed poor color stability due to the fact that they are manufactured in layers with incomplete bonding, large amount of residual monomer leakage during post‐curing processes after printing and low polymerization rate [20]. 

The CAD-CAM milled group was comparatively superior in color stability to the conventional acrylic resin materials. This is due to changes in the optical properties of the resin materials caused by water sorption as conventional heat polymerized PMMA is liable to insufficient pressure or excessive heating during exothermic polymerization reaction which can cause excessive amount of residual monomers leach out [9], and poor surface roughness [10, 11]. 

In addition, higher probability of processing defects such as micro-porosity [12] might occur during the curing cycle of heat polymerized PMMA group which weakens polymer chains and eventually leads to water sorption without hygroscopic expansion [13]. This directly minimizes the consistency of color in base materials for dentures [44]. This study revealed that in the denture cleanser solution, the CAD-CAM milled group showed a lower color change compared to the 3D-printed and the heat polymerized PMMA group (*P* > .001). This finding was consistent with the results of Alfouzan et al. [44], Gruber et al. [56], and Dayan et al. [22] The present results, however, differed from those of Jain et al. [62] This contradiction could be linked to variations in the chemical composition, the method of action as well as the manufacturer of the denture cleansing tablet tested. It was obvious that, color changes of the specimens immersed in denture cleanser were greater than the changes in △E observed in thermocycled specimens. This may be caused by the high degeneration rate of sodium perborate which present in effervescent denture cleansing tablets to alkaline peroxide form after dissolution in water during cleaning [35]. 

When sodium perborate is dissolved in water, it produces sodium metaborate, hydrogen peroxide, nascent oxygen, and an alkaline peroxide solution. In addition to oxygen-releasing components such sodium perborate or percarbonate, the peroxide solution also contains alkaline detergents that lower surface tension. There is a mechanical and chemical cleaning action of releasing oxygen bubbles [35, 36]. 

High degree of surface roughness, high peroxide content, high PH level and oxygenation in alkaline solutions were reported to have a major negative influence on the color stability of denture acrylic resins [43]. On the other hand, acrylic resin discoloration which occurs during thermocycling, is caused by only two factors; either temperature or humidity change leading to volumetric contraction or expansion of materials [59], alteration of the polymer’s matrix and decomposition of material [28]. 

Limitations of the present study include that the exact simulation of oral environment could not be achieved. Oral hygiene, salivary PH and quantity of saliva may affect the result. Clinical studies are required while testing different manufacturers of each type of denture base resin.

## Conclusions

The study’s findings led to the following conclusions:

1. The CAD-CAM milled resin in CD production exhibits greater dimensional accuracy compared to both the 3D printed resin and the traditional heat polymerized PMMA. Among the three systems evaluated in this study, the 3D printed resin had the lowest dimensional accuracy.
2. CAD-CAM milled resin had the highest color stability, while the 3D- printed resin had the lowest Color Stability and conventional Heat Cure PMMA lies in between them both.
3. Color changes resulting from immersion in denture cleansers is greater than that from thermocycling.

## Data Availability

The datasets produced and examined during this study could be obtained from the corresponding author upon a reasonable request.

## Abbreviations

CAD-CAM: Computer-aided design and computer-aided manufacturing
3D: 3-dimensional
CDs: Complete dentures
STL: Standard tessellation language
CNC: Computer numerically controlled
ISO: International Organization for Standardization
IQR: Inter-quartile range
SD: Standard deviation
PT: The perceptibility threshold
AT: Acceptability threshold
